# Lifestyle interventions to reduce non-communicable disease risk in female secondary school teachers of Islamabad; a quasi-experimental mixed-methods protocol

**DOI:** 10.3389/fpubh.2025.1641499

**Published:** 2025-09-12

**Authors:** Zoha Imtiaz Malik, Shaheer Ellahi Khan, Abdul Momin Rizwan Ahmad

**Affiliations:** ^1^Department of Public Health, Health Services Academy, Islamabad, Pakistan; ^2^Department of Human Nutrition and Dietetics, NUST School of Health Sciences, National University of Sciences & Technology (NUST), Islamabad, Pakistan; ^3^Institute of Health Policy, Management, and Evaluation, Dalla Lana School of Public Health, University of Toronto, Toronto, ON, Canada; ^4^Department of Health Sciences, University of York, York, United Kingdom

**Keywords:** non-communicable diseases, lifestyle modification, female teachers, secondary schools, anthropometrics

## Abstract

**Background:**

Non-Communicable Diseases (NCDs) are rapidly increasing globally, including Pakistan. Female school teachers form a vital yet often ignored population sub-group in terms of NCDs prevalence and risk factor clustering. Despite growing burden of lifestyle associated NCDs, there is limited data on workplace based lifestyle interventions, particularly in school settings.

**Objective:**

The proposed study aims to evaluate the effectiveness of a lifestyle intervention on NCDs risk factors and their awareness in female secondary school teachers, as well as to identify the barriers and facilitators to its adoption.

**Methods:**

A quasi-experimental mixed methods study will be conducted in public secondary schools of Islamabad city, Pakistan, including both an intervention and control group. The study will be divided into three phases; baseline assessment, intervention development and implementation, and an end-line assessment. Comparisons will be made between control group and intervention groups, as well as pre and post intervention. Following the end-line assessment, in-depth interviews will be conducted on a sample of teachers to explore the barriers and facilitators to adoption of lifestyle changes. A total of 130 teachers, recruited through purposive sampling, will be divided into 2 groups of 65 in intervention and control groups each. The intervention will span 9 months, and consist of bi-weekly education and training sessions on lifestyle modifications. The sessions will include topics relevant to nutrition and physical activity, and take home resources such as sample menus, list of healthy food options etc. Primary outcomes of the study include anthropometric measurements including weight, body mass index (BMI), waist and hip circumference (WHC), waist to hip ratio (WHR). Secondary outcomes will include knowledge and awareness of NCDs and their associated risk factors.

**Discussion:**

The study aims to reduce NCDs risk factors among female teachers by incorporating tailored lifestyle interventions including both nutrition and physical activity. This study intervention can help promote overall health of working females, improve workplace wellness and foster a culture of health promotion and disease prevention in educational settings. The findings will facilitate policy-makers to generate sustainable health strategies to empower teachers to take control of their health, and build a healthier educational workforce.

## 1 Introduction

Non-communicable diseases (NCDs) are rapidly increasing in prevalence globally, and account for 74% annual deaths worldwide (1). Diabetes, hypertension, and cardiovascular diseases are among the top most prevalent NCDs, with diabetes effecting 529 million people around the world (2). Hypertension currently impacts 626 million women and 652 men (3), while cardiovascular diseases cases around the world have risen to 523 million (4). The South-Asian region, like the rest of the world, is also heavily burdened with NCDs, where these diseases cause approximately 9 million annual deaths (5). Pakistan is experiencing growing NCD related morbidity and mortality rates, and 58% of annual deaths can be attributed to these diseases (6). Over the years, research has evidenced that majority of these NCDs emerge from an array of risk factors, both modifiable and non-modifiable. The modifiable factors include, unhealthy diet, sedentary lifestyle, tobacco use, and alcohol consumption, whereas age, gender, genetics, ethnic group are classified as non-modifiable (7). Gender plays a crucial role in NCD prevalence and associated complications. Women are more prone to developing NCDs due to increased clustering of risk factors in this gender group (8). It has been reported that 11.4% women experience a clustering of both types of risk factors. Additionally, women are more prone to remain un-diagnosed, resulting in increased complications and adverse effects (9). Women in low and middle income countries have NCD prevalence rates of 82%. Additionally, NCD associated maternal deaths reached a prevalence of 44% (10). Pakistani women report an increasing prevalence of NCD risk factors (12.5%), and diabetes (9.1%) (11).

Women in the workforce, despite working in the same environment as their male counterparts, have a heightened risk of developing non-communicable diseases due to their biological differences. Additionally, carrying both workplace and traditional domestic responsibilities puts a toll on women’s psychological health which further aggravates various pathways that lead to development of metabolic irregularities, as suggested by a systematic review conducted by Idris et al. (12). This is why employed females report 1.05 times higher risk of having multi-morbidity as compared to unemployed females (13). Work related stress has been reported to elevate the chances of developing type 2 diabetes in women as much as 12%. Increased stress levels contribute to higher cortisol levels, leading to insulin resistance, raised blood glucose levels, and higher visceral obesity, all of which contribute to diabetes mellitus and other NCDs. Additionally, increased working hours are associated with a greater risk of developing lifestyle risk factors, such as poor diet, physical inactivity, smoking and alcohol consumption, all of which further amplify chronic diseases probability (14).

Recently, a large body of evidence has depicted positive results of lifestyle modification on reducing non-communicable diseases risk factors in both men and women. Physical activity and improved diet have a significant impact on reducing weight, decreasing overall fat intake and increased fiber intake, all of which help reduce lifestyle risk factors (15). Pairing preventive cardiovascular disease screening with lifestyle interventions focused on dietary and physical activity modifications produced small but long-term health benefits in women, especially those with preeclampsia (16). Whether diet or physical activity or both produce the greatest impact on reducing non-communicable disease risk factors depends on a number of factors such as the type of diet, intensity and duration of physical activity and other physiological conditions such as pregnancy, post-partum or lactation (17). Lifestyle interventions including both nutrition and physical activity components can produce modest health impacts in as little as 6 weeks. Improvements were seen in the body mass index (BMI), arm, chest, waist and hip circumferences, body far percentage, and blood glucose levels after a one and a half month aerobic training and nutrition education community program targeted toward women aged 50 years and above (18).

Teachers working at primary and secondary schools depict similar lifestyle behaviors and have below par health status. About 80.9% of female school teachers lacked adequate nutrition knowledge, and 17.6% were obese (19). Evidence has shown that improving the lifestyle related knowledge and behaviors in teachers can directly and positively impact the nutritional and physical activity associated behaviors in students (20). Additionally, studies have found teachers to have a higher prevalence of unhealthy lifestyles and overall a poorer health status, as compared to medical professionals or entrepreneurs (21). Furthermore, poor health in teachers then leads to increased absenteeism and even early retirement as the quality of life further deteriorates. Evidence has depicted a positive impact of physical activity interventions on reducing stress, and employee turnover, while improving health outcomes. This ultimately increases the overall education system productivity and reduces healthcare costs (22).

A study carried out on Nigerian female teachers reported a significant decrease in the prevalence of hypertension, diabetes mellitus, and hypercholesterolemia following a 3 month intervention that included scheduled exercise sessions and dietary control recommendations (23). An Indian study conducted in teachers found significant increases in exercise, fruits and vegetable consumption, with a simultaneous reduction of sodium, fat, red meat and processed food intake, following a lifestyle education intervention (24). Evidence suggests that female teachers have a considerable level of unhealthy dietary patterns and these are significant implications of their nutritional habits on the dietary choices and behaviors of their students (25). Hence, promoting healthy food intakes among teachers through different interventions can not only improve their own health outcomes but also positively influence their student’s lifestyle perceptions and habits.

There is a large body of work that demonstrates the impact lifestyle interventions have on reducing the risk factors of various NCDs, however, limited data is available on the participant’s perceived barriers and facilitators to adopting a healthier lifestyle, as evidenced by a systematic review on the subject (26). A meta-analysis of qualitative studies explored the supporting and constraining factors toward the adoption of lifestyle interventions in pre-diabetics. One of the key highlighted theme was the internal struggle of making these changes, which participants reported to be overwhelming and daunting to incorporate in their daily life (27). Another study implemented lifestyle interventions at the workplace and reported their positive impact which led to a significant decrease in health related stress, thereby also improving work performance (28). A study, focused on increasing lifestyle intervention adoption in obese women, reported key findings to tailor a behavior change program; incorporating holistic health model in education sessions, administer personalized feedback and individualized goal setting to remove barriers, and focus on building sustainable lifestyle changes that can be continued even after the program ends (29). Sustainability of lifestyle changes can be ensured when participants receive adequate support from their healthcare team as well as their social circle including close family and friends. Group settings provide a high degree of relatedness with others as well as feeling supported by a community to make changes in lifestyle. Constant participant feedback in the form of in-depth interviews or focus group discussions provide insight into their perceived barriers and facilitators in the process, and can form the basis of developing successful lifestyle interventions (30).

There is a major research gap in Pakistan regarding the NCD epidemic among women. In recent years Pakistan has managed to contribute only 1.7% to the global female NCDs bibliometric analysis. A lack of evidence based research which can be further translated into policies and programs to overcome the NCD epidemic is majorly missing in Pakistan’s response to NCD prevention and control. Working females particularly teachers have an even greater risk of NCDs as they are consistently exposed to stressful situations and a lack of time, both of which have established associations with the occurrence of non-communicable disease risk factors. Targeting a lifestyle intervention to this population group can help promote a healthier lifestyle in female educators, which is also seen to indirectly improve the health behaviors of their pupils, by acting as role models. Improved health status of teachers is also linked with improved productivity and reduced health costs. This study can help prioritize, develop and implement women specific recommendations to reduce NCD prevalence in this gender group.

Hence, the overall aim of this study is reducing the non-communicable disease (NCDs) risk factors and improving knowledge and practices regarding NCDs and their risk factors among female public secondary school teachers in Islamabad. The specific objectives are:

- To carry out baseline assessment of NCDs risk factors in female public secondary school teachers in Islamabad.
- To determine baseline knowledge, attitudes and practices regarding NCDs and their risk factors in female public secondary school teachers in Islamabad.
- To adopt and implement a lifestyle (nutrition and physical activity) intervention to reduce the burden of non-communicable disease risk factors and improve their associated knowledge, and practices in female public secondary school teachers in Islamabad.
- To carry out an end line assessment to determine the feasibility of up-scaling the intervention and explore the barriers and facilitators toward its adoption in female teachers of public secondary schools.

## 2 Methods and analysis

### 2.1 Study design

The study will be a mixed methods research, employing both quantitative and qualitative aspects in data collection and analysis. The reason for using a mixed methods study is because quantitative and qualitative researches are concerned with different questions and research paradigms. While quantitative research focuses on numbers and proportions, qualitative research delves deeper into the how’s and why’s of a research problem. Quantitative research is influenced by ‘positivism’, assuming that any problem can be measured and quantified, irrespective of its nature and underlying causes. Whereas, qualitative research employs interpretivism, which assumes that each individual has a different world view and that everyone experiences a reality in a different way. Therefore, mixed methods studies are imperative in bringing together multiple ways of looking at a research question and providing a deeper insight into the severity, underlying causes, and potential solutions to these problems (31). Furthermore, when talking about lifestyle interventions or lifestyle change programs, there remains insufficient data on the facilitators and barriers to adopting the lifestyle modification guidelines. This can be addressed by employing a mixed-methods approach incorporating strategies to identify the causes of participant engagement and adherence to lifestyle interventions, and then coming up with solutions to improve these aspects (32). Our mixed methods study can help bridge this gap in literature and provide important information needed to increase the success rate of lifestyle interventions.

Additionally, the study will be a quasi-experimental study design, specifically the difference-in-differences (DID) quasi experiment, with one intervention and one control group. The DID analysis helps to determine the impact of the intervention by measuring the change in the outcome variable over time and doing a comparative analysis between the intervention and control groups. It helps evaluate the existence and strength of a causal relationship, especially in situations when a randomized controlled trial cannot be implemented, either due to ethical reasons or lack of resources to carry out these trials. For the DID design to accurately determine the causal relationships, it assumes that both the control and intervention groups adhere to ‘parallel trends’ to each other if the intervention did not exist (33). Difference in difference studies are used frequently to assess the intervention impact in a population group, therefore it aligns with our study’s objectives. In these studies we compare the difference in outcomes between the intervention and control groups, which depicts the factors that change overtime within these groups. We analyze two differences in these experiments; the first difference is the before and after comparison in the intervention group, which tells us what factors remain the same over time despite the implemented intervention. The second difference is between the control and intervention group, which tells us what factors were influenced or changed by the intervention over time.

Our study will include a baseline assessment of all study variables, in both intervention and control group, to address the parallel trends assumptions of the DID study design. This will help establish baseline equivalence across both groups on all variables prior to intervention implementation. Although, the study only has one pre-intervention time point, this baseline similarity will strengthen the plausibility of parallel trends. Furthermore, any observable differences will be statistically adjusted for through regression modeling. This will help control any potential confounding factors in the study. For substantial baseline imbalance across key covariates, propensity score weighting will be applied within the DID framework to enhance internal validity. This approach is widely used in DID quasi experimental design studies where multiple pre-intervention points or randomization is not feasible (34).

### 2.2 Phases of the study

**Figure 1:**
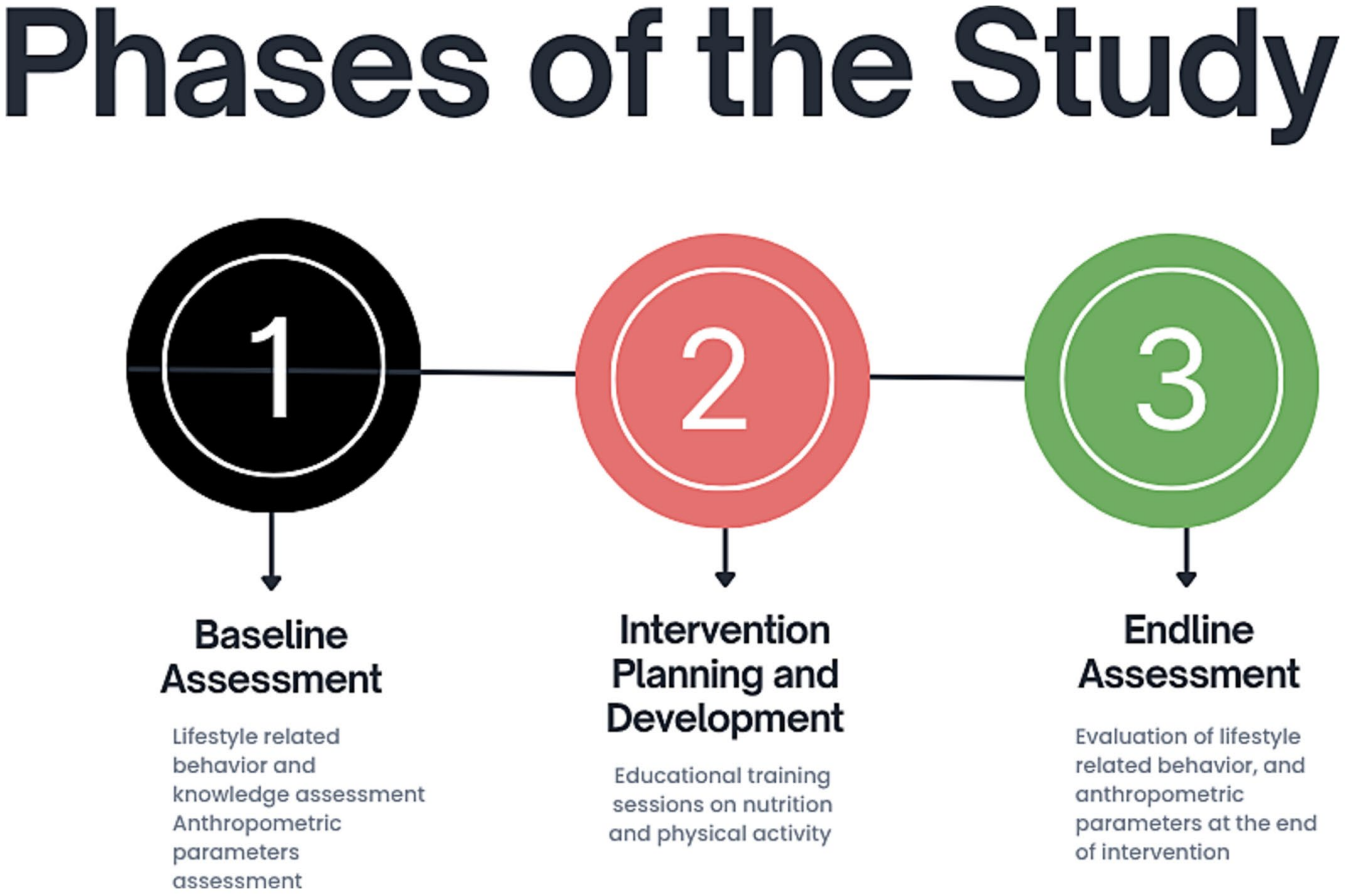
Phases of the Study: this figure outlines the three main phases of our research, including a baseline phase, followed by intervention development and finally an endline assessment.

The study will be divided into 3 main phases, as shown in Figure 1:

- Phase 1: Baseline assessment of female teachers of public secondary schools.
- Phase 2: Intervention adaptation and implementation in female teachers of public secondary schools.
- Phase 3: End-line assessment in female teachers of public secondary schools.

#### 2.2.1 Phase 1: baseline assessment of outcome variables of the study participants

The baseline assessment of the following variables will be conducted:

- Behavior and knowledge assessment—these will include questions on dietary intake, physical activity, awareness on lifestyle risk factors of NCDs. These were included in the study as 40% of all cancers and 80% of heart diseases can be attributed to behavioral risk factors, four of which are most prevalent; smoking, alcohol consumption, physical inactivity and unhealthy diet. However, there remains a lack of surveillance and reporting of these risk factors at local, national and international levels. If recognized and managed early, the NCD burden and severity could be reduced by a great extent (35). The modern day strategy to manage NCDs is based on the risk factor management of these diseases, particularly the modifiable or behavioral risk factors. It involves making changes at individual, societal, national, and international levels by increasing awareness regarding the behaviors leading to NCD development and developing policies to control the occurrence of these risk factors among communities, such as tobacco control laws, regulation of sugar sweetened beverages, development of physical activity opportunities at work and educational institutes (36). Our study aims to include the mentioned behavioral risk factors and determine the impact a lifestyle modification intervention can have in reducing them. This can have policy implications, as in case of favorable results the proposed lifestyle modification can be implemented at the local or regional levels.
- Anthropometric or physical measurements—We have included body mass index (BMI), waist circumference (WC), hip circumferences (HC), waist to hip ratio (WHR), and waist to height ratio (WhTR) in our study to determine obesity risk and levels in the study population. Obesity is a vital risk factor for non-communicable diseases and contributes to metabolic disturbances seen in various NCDs. Over the years, body mass index (BMI) has been used to determine the level of obesity in individuals, but recently other anthropometric measurements have been considered to classify obesity. These include waist circumference (WC), hip circumferences (HC), waist to hip ratio (WHR), and waist to height ratio (WhTR). All these measurements either independently or coupled with BMI have been seen to accurately estimate abdominal obesity and visceral fat, both of which are risk factors for NCDs. Studies have concluded BMI, WC and WHtR as reliable anthropometric estimates of obesity which can then be used to accurately detect obesity and subsequent NCDs, particularly in women as abdominal fat is associated with greater risk of developing NCDs in the female population (37). An Indian study aimed to assess the association between anthropometric measurements and NCD risk and found that in adults aged more than 45 years, are 61 and 98% more likely to develop NCDs and multi-morbidities if they have high waist to hip (WHR) and high waist circumference (WC), respectively (38). Therefore, employing anthropometric measurements as an assessment factor for NCDs can help provide a clear picture of the level of NCD development risk in the population.

#### 2.2.2 Phase 2: intervention development and implementation

The intervention phase will involve exposing the intervention group to nutrition and physical activity knowledge in the form of educational sessions, which will be divided into 12 sessions. Three sessions will be given each month for the first 6 months, and two sessions will be given each month for the last 3 months. The intervention sessions will be delivered by trained health professionals including registered dietitians, and physical fitness experts. The intervention will be delivered in a group format where the participants will receive joint counseling. However, participants will be encouraged to seek individualized guidance during or after sessions, to address any personal concerns. Individualized diet plans may also be administered to participants with special needs or multiple risk factors. This will ensure tailored support within a group-based framework.

- Nutrition intervention—will comprise of bimonthly education and counseling sessions on healthy food choices, identifying and reducing intakes of unhealthy foods, equipping participants to prepare healthy meals from the foods available at home, self-monitoring of eating habits and NCD risk factors, and incorporating nutrition guidelines from Pakistan Dietary Guidelines for Better Nutrition (PDGN) into daily life. A total of 12 sessions will cover important nutrition topics to increase participant awareness of healthy diets and individual diet plan provision will help them guide them in their daily nutrient intake. The topics for these sessions are given below:
- Physical activity intervention—will include physical activity education and training sessions as per the Pakistan Dietary Guidelines for Better Nutrition (PDGN) recommendations, and WHO physical activity guidelines. One session per 15 days will be done and participants will be guided on how to incorporate physical activity in their daily routine. Aerobic exercises such as walking and jogging will be promoted and the use of pedometers will be encouraged to count daily steps. Each participant will be advised to walk at least 30 min every day for a minimum of 5 days a week as per WHO guidelines.

**Figure 2:**
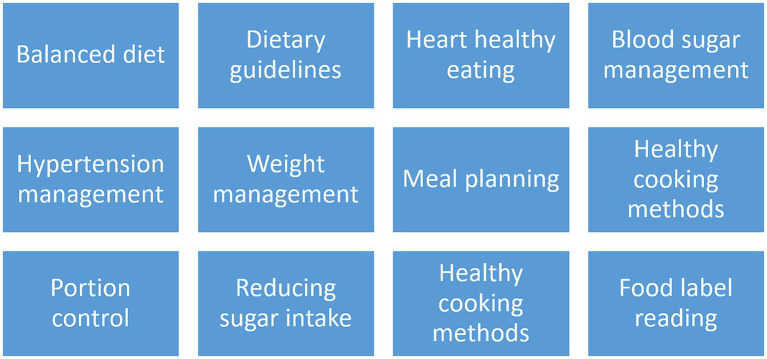
Nutrition intervention session topics: this figure covers the weekly nutrition education sessions tailored to female secondary school teachers, as part of the proposed intervention to reduce NCDs risk.

**Figure 3:**
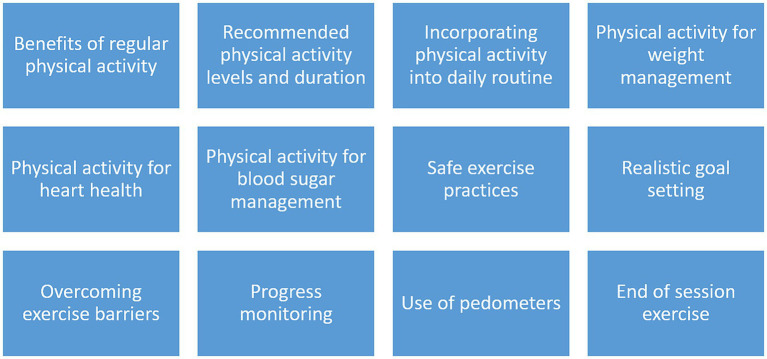
Physical activity intervention session topics: this figure includes topics relevant to physical activity promotion in female teachers and reducing barriers to physical activity as part of a tailored lifestyle education intervention.

The topics for these sessions are given in Figures 2, 3.

Nutrition interventions will be designed in direct alignment of the Pakistan Dietary Guidelines for Better Nutrition (PDGN). These guidelines are specifically designed to meet the nutritional needs and dietary patterns of the Pakistani population. Nutrition sessions will be tailored according to the following key PDGN sections:

- Nutritional status of adults (19–60 years)
- Relationship between diet and disease
- Traditional and fast foods
- Salt consumption
- Glycemic index of foods
- Nutrition labeling
- Overweight and obesity
- Physical activity and lifestyle behavior
- Pakistan dietary guidelines for better nutrition
- Food and serving size for adults (19–60 years)
- Sample menu for adults (19–60 years)
- Dietary Messages

The physical activity sessions will include recommendations from the PDGN’s Annexure-XIII: Recommendations on physical activity for different age groups, which have been developed considering the Pakistani population’s contextual factors. Furthermore, the WHO’s physical activity guidelines will also be used as a reference to shape the intervention according to the study population, in order to ensure the global physical activity standards for NCD prevention. Additionally, thrice weekly group exercise sessions will be arranged to promote physical activity guidelines adherence.

Intervention fidelity will be ensured through content validation of the session material through subject experts who will ensure the intervention is relevant to study objectives. Additionally, a session plan will be developed and adhered to, to ensure all material is delivered timely and accurately. Standardized checklists and attendance logs will be maintained for each session to assess content delivery and participant engagement. Furthermore, study participant adherence will be ensured through weekly text message check-ins in participant WhatsApp groups. Secondly, participants will be asked to maintain bi-monthly dietary logs or diaries. Moreover, sessions will be scheduled according to teachers’ availability in consultation with school administration to promote their participation.

The control group will go about their usual routine and will receive no nutrition or physical activity related sessions or educational material during the study period. Once the study has concluded they will be given lifestyle intervention sessions as an ethical consideration. However, to ensure consistency in data collection and monitoring, the control group will undergo standardized follow-up assessments at the same time frames as the intervention group (baseline and endline). Additionally, monitoring visits will be conducted at the control group schools to ensure engagement, minimize attrition, and warrant consistent study conditions. Lastly, fidelity checks will be conducted during data collection, by research supervisors, to ensure standardized administration of questionnaires and anthropometric measurements.

#### 2.2.3 Phase 3: end-line assessment of outcome variables

Evaluation phase to assess the impact of the intervention in each group as well as to compare the impacts of the interventions with each other. This phase will also include in-depth interviews of participants to identify the facilitators and barriers that played a role in either helping them follow the dietary guidelines given or not be able to do so. Additionally, the reasons behind their knowledge, attitudes and practices regarding NCDs and their risk factors will be explored via focus group discussions.

### 2.3 Study area

The study area will be Islamabad city, and schools from the city will be included. The reason for choosing Islamabad city is because the prevalence of non-communicable diseases is higher in the urban areas as compared to the rural areas of Pakistan as reported by the Pakistan Demographic and Health Survey 2017–18. The survey found a direct association between education and wealth status and non-communicable disease and risk factor prevalence, with 40% women with the highest wealth status being overweight as compared to 16% women of low socio-economic status. Among the provinces and cities, Islamabad reported the highest prevalence of overweight and obese women (68%). Therefore, female educators in Islamabad form a vulnerable group in terms of NCD development and an intervention targeting these women can help reduce their risk of developing these diseases later in life.

### 2.4 Duration of study

The study will be conducted over a period of 17 months and the timeline will be as follows:

Phase 1 - Baseline data collection – 02 months.

Phase 2 - Intervention development and implementation

- Phase 2a - Planning and adaptation of interventions − 02 month
- Phase 2b - Pre-testing the intervention − 02 months
- Phase 2c - Intervention implementation − 09 months

Phase 3 - Evaluation of intervention impact − 02 month.

### 2.5 Data sources

This study will involve primary data collection and employ the questionnaire method of gathering data from participants. Participants who have given their consent to be a part of the study will be asked to fill in the adapted and validated questionnaires containing different variables of interest.

### 2.6 Study population

The study population will be all female teachers employed in the public secondary schools of Islamabad.

### 2.7 Sampling technique

Study will be a 2 stage cluster sampling, as the sampling procedure will be divided into 2 steps.

#### 2.7.1 Stage 1: selecting schools

- Defining clusters: all public schools within Islamabad city will be considered clusters.
- Sampling frame: list of all public schools in Islamabad city will be the sampling frame and will be obtained from the education department.
- Number of schools selected: according to Pakistan Education Statistics report (2021–22) 15 teachers on average in each school – 130/15 = 8 schools selected via purposive sampling. Purposive sampling was chosen to select schools based on practical considerations such as administrative permissions and accessibility, institutional cooperation, and the practicality of implementing a lifestyle intervention in real-world school settings. This will enhance the study’s contextual relevance, and help understand the feasibility of future up-scaling within similar environments (39). Employing purposive sampling may limit the study’s applicability to other population groups such as private or rural school teachers. However, utilizing purposive sampling will help prioritize contextual comprehension and intervention feasibility at a deeper level. This is consistent with implementation research frameworks that prioritize situational understanding over statistical representativeness (40).

#### 2.7.2 Stage 2: selecting teachers within schools

- Sampling frame: list of teachers from each selected school will be obtained from the school’s administration and will serve as a sampling frame.
- Selection of participants – all female school teachers will be screened for NCD risk factors using the WHO STEP wise Approach to NCD Risk Factor Surveillance (STEPS) tool, which includes both behavioral assessment (Step 1) and anthropometric measurements (Step 2). Teachers identified in the screening process to meet predefined NCD risk criteria, will be considered eligible for the study and will be selected via simple random sampling such as lottery method to be enrolled in either intervention or control arm.

### 2.8 Sample size calculation

The sample size is calculated on the basis of BMI reported from a similar study conducted in India (41) using the formula: $n=[2\times (\sigma^{2})\times {\left(Z₁-\alpha /₂+Z₁-\beta \right)}^{2}]/d^{2}$

Where SD = 0.96.

Alpha = 0.05.

Power of study = 80%.

Effect size = 0.5.

Non-response rate = 10%.

Sample size = 65 per group and total sample = 130 teachers (both groups).

### 2.9 Sample recruitment: inclusion and exclusion criteria

Inclusion and exclusion criteria for the study is shown in Table 1. Female teachers of selected public secondary schools meeting the inclusion criteria will be approached and those consenting to be a part of the study will be recruited.

**Table 1:** Inclusion and exclusion criteria.

Inclusion criteria	Exclusion criteria
Females aged 30 to 45 years of age.	Female teachers who are undergoing any medicinal, herbal, or nutritional therapies.
Females who have not been medically diagnosed with a non-communicable disease.	Pregnant and breastfeeding females.
Females who have one or more of the non-communicable disease risk factors: BMI > 25 kg/m^2^ Waist circumference > 88 cm Fruit and vegetable intake < 5 servings per day Physical activity < 150 min/week	Female teachers who will not be staying in the school for the next 9 months

### 2.10 Data collection techniques

Data collection will be done using validated and pre-tested questionnaires, designed specifically to obtain data on non-communicable disease risk factors and dietary and physical activity adherence levels. These questionnaires will be administered through face to face surveys where the researcher will distribute the questionnaires to the study participants and guide them on how to fill them. Researcher will take the body measurements of the subjects for anthropometric data and glucometers and cholesterol test kits will be employed to get biochemical parameter data.

The questionnaires will be administered in the 1st and 3rd phase of the study to get a baseline and evaluative risk factor assessment. Female teachers will be approached in their place of employment and once they have given consent to be a part of the study they will be asked to fill in the questionnaires. The participants will be briefed prior to questionnaire administration so that they may be able to understand what is being asked and how to best answer it.

### 2.11 Data collection tools

The following two tools will be used to collect data:

#### 2.11.1 ‘WHO STEP wise approach to non-communicable disease risk factor surveillance (STEPS) questionnaire’

The STEPS questionnaire is divided into 3 sections:

- STEP 1 – demographic and behavioral risk factor assessment such as age, gender, education, employment status, as well as health assessment such as dietary intake data, physical activity assessment, and disease, specifically NCD history.
- STEP 2 – anthropometric assessment including body measurements such as weight, height, waist circumference, body mass index (BMI).
- STEP 3 – biochemical assessment including blood tests for glucose, cholesterol and lipid levels.

In our study, the questionnaire will be adapted to include only the first two steps and not biochemical assessment due to funding restraints.

#### 2.11.2 General knowledge and attitudes related to non-communicable diseases (GK), NCD behavioral risk factors (RF), and different NCDs

It was developed and validated by Yenit et al. will be adapted (42). It assess knowledge across three NCDs: diabetes mellitus, hypertension, and cardiovascular diseases (CVDs). Each disease section has its own maximum knowledge score: 22 points for diabetes, 10 points for hypertension, and 22 points for cardiovascular diseases. Additionally, overall knowledge scores will be interpreted using tertile cut-offs. Scores less than 33rd percentiles will be considered low knowledge, 33^rd^ to 66^th^ percentile will be moderate knowledge and more than 66th percentile will be termed high knowledge scores. The general knowledge and attitudes regarding NCDs set the basis for developing them in later years. Studies have focused on collecting data on different population group’s knowledge regarding NCDs but there remains a gap in associating this knowledge with the risk of developing NCDs. Our study aims to address this by associating the level of NCD related knowledge and attitudes with the behavioral and anthropometric risk factors of NCDs. Furthermore, our study’s population is urban female educators and gauging their knowledge and attitudes can help us get a deeper insight into the NCD related knowledge in urban women. A Malaysian study found that rural respondents had better NCD attitudes and practices as compared to the urban population, whereas there were no significant differences in NCD knowledge. Additionally, despite urban population having greater knowledge regarding NCDs, their attitudes and practices remained unsatisfactory (43). This calls for NCD awareness interventions in the urban population groups, which is also the basis for our study’s intervention. A Zimbabwean study found 65% of the study participants to be knowledgeable about NCDs and 81% reported good perceptions about these diseases. These results were significantly influenced by age, gender, and education level of respondents, with females being 1.8 times, those ages 40 years and above being 0.1 times and those with primary education level being 0.2 times more likely to have better perception of NCDs (44).

#### 2.11.3 National Knowledge, attitudes and practices survey on non-communicable diseases

The data for the third outcome variable will be collected using the ‘National Knowledge, Attitudes and Practices Survey on Non-Communicable Diseases’ developed and validated by Demaio et al. (45) will be adapted to assess the participants’ knowledge, attitude and practices regarding the NCDs and their risk factors at both baseline and end-line assessment. It uses a combination of likert scale, true/false, and multiple choice questions to assess knowledge, attitudes and practice scores. The knowledge items are scored as correct = 1 and incorrect/do not know = 0. Attitude items are scored on a likert scale as strongly disagree = 1 to strongly agree = 4, and are coded in reverse where necessary. Practice items are scored as healthy behavior = 1 and unhealthy/risky behavior = 0. Domain specific scores are summed and categorized in low, moderate and high based on established tertiles or study specific score distribution.

#### 2.11.4 Focus group discussions

Focus group discussions will be carried out with female teachers. An interview guide will be developed and validated to collect data from participants regarding the barriers and facilitators for their adoption of lifestyle changes/adherence. A total of 4 groups comprising of 6–8 participants each will be conducted. Therefore, 24–32 participants will be selected for focus group discussions. Both intervention and control group participants will be stratified to get differential perspectives on the intervention. Additionally, 10–12 in-depth interviews will be conducted with school principals and teachers. This will provide deeper insight into the barriers and facilitators to lifestyle change at the individual and administrative levels. Participants will undergo purposive selection to ensure maximum variation in age, teaching experience, socio-economic status, and other socio-demographic variables. The final number of focus groups and interviews will be determined by data saturation point, where no further themes emerge.

### 2.12 Study outcomes

#### 2.12.1 Primary outcomes

The primary outcomes of the study include body mass index (BMI), dietary and physical activity behaviors, and NCD awareness (44).

#### 2.12.2 Secondary outcomes

The secondary outcomes for the study include anthropometric measures including waist circumference (WC), hip circumferences (HC), waist to hip ratio (WHR), and waist to height ratio (WhTR), and NCD risk factors awareness (45).

### 2.13 Conceptual framework

The study will incorporate the Socio-ecological model (SEM) in its qualitative inquiry to identify the barriers and facilitators to the adoption of lifestyle modification. The model provides a holistic approach to understand how personal, inter-personal, social, organizational, and physical environments plays a role in shaping one’s health related behavior. This model is also helpful in navigating health policies, as it highlights the components that most influence health and lifestyle choices (46). The model divides the determinants of health behavior into 4 levels; individual factors such as knowledge, inter-personal factors including relationships with family and peers, community factors such as access to healthy foods, and policy level such as access to gyms at workplaces. Using the SEM model helps target multiple levels of health behavior and can identify which ones have the most impact, as either a barrier or a facilitator, in adopting healthier lifestyle beyond the individual level (47).

In addition to the SEM, the intervention will be guided by the Trans-Theoretical Model (TTM). The TTM helps guide participant engagement and facilitates behavior change. The model views behavior change as dynamic process spanning over 5 distinct stages including, pre-contemplation, contemplation, preparation, action, and maintenance. The TTM states that for an individual to achieve sustainable behavior change, he/she must go through each stage or else there may be a significant risk of relapse. Tailoring the interventions according to the participants’ degree of readiness increases chances of success. The TTM further allows for individualized and stage appropriate guidance which enhances intervention effectiveness and sustainability (48). Additionally, in group based interventions, TTM has proven to be effective as it facilitates peer support while simultaneously allowing for individual reflection. TTM acknowledges that behavior change is gradual and non-linear, therefore it is flexible, theoretically grounded, and participant focused. These attributes help improve the relevance and implementation feasibility of public health interventions (49).

### 2.14 Data analysis plan

**Figure 4:**
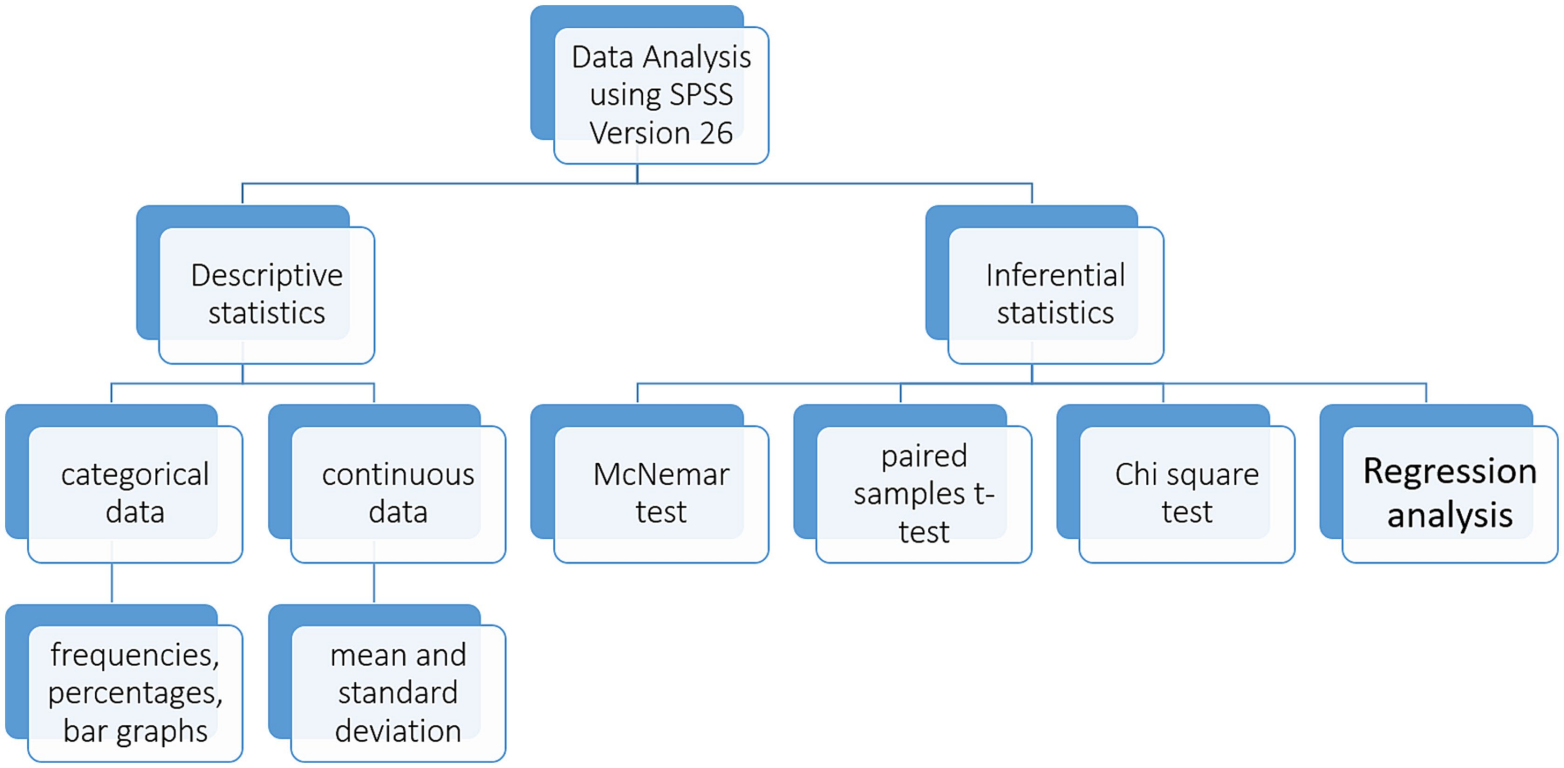
Quantitative data analysis plan: this figure includes a summary of the descriptive and inferential quantitative data analysis plan.

The data collected will be analyzed via SPSS version 26. The confidence interval will be set at 95%, hence *p* value of less than 0.05 will be considered significant in the findings. The data analysis plan is shown in Figure 4.

#### 2.14.1 Descriptive statistics

Data will be analyzed using frequencies, percentages to report the prevalence and proportion of NCD risk factors and their associated knowledge and attitudes in the study population. A comparative analysis will be done between the baseline and end line data to study the influence of our study’s intervention in reducing the NCD risk factors and improving the knowledge and practices regarding them and their contributing behaviors.

#### 2.14.2 Inferential statistics

- Paired samples t-test will be used to compare the pre and post intervention values of continuous variables
- McNemar test will be used to compare the pre and post intervention values of categorical variables
- Chi-square test will be applied to determine associations between different variables
- Regression analysis will be conducted to estimate the direction and strengths of relationships between independent and dependent variables
- To adjust for potential confounders such as socio-demographic variables, multivariable regression models will be employed. Variables such as age, household income, education level, will be included as covariates in the DiD analysis. This will help adjust for baseline differences between control and intervention groups, ensuring more accurate attribution of the intervention’s effects. This further enhances the internal validity of the study via limiting confounding bias.
- To control the risk of type I error, statistical corrections will be applied for multiple comparisons, where appropriate. This will include Bonferroni correction and/or false discovery rate (FDR) correction.

#### 2.14.3 Qualitative data

Qualitative data analysis will be done in multiple structured steps:

1. Software: All interview transcripts will be managed in NVivo 12.
2. Coding Process: Two researchers will independently develop an initial codebook using a combined inductive–deductive approach. After coding a 10 percent sample of transcripts, the codebook will be refined through discussion.
3. Inter-coder Reliability: We will calculate Cohen’s *κ* on a second 10 percent sample of transcripts, iterating until κ ≥ 0.80 is achieved. Discrepancies will be resolved by consensus or by a third senior investigator.
4. Theme Validation: Final themes will be reviewed in a stakeholder workshop with two participants from each school to confirm interpretive validity.

A six step framework will be used to extract themes from the data collected through in-depth interviews:

1. Reading and familiarization with collected data
2. Identifying codes from the data
3. Reviewing the codes to develop themes from the data
4. Reviewing the generated themes
5. Classifying the themes
6. Reporting and summarizing the themes

Thematic analysis will help identify the recurring themes surrounding NCD risk factors, their knowledge, attitude and perceptions in the study population. Thematic analysis of data will emphasize on how the repondent’s view and ideas about their health, risk factors and NCDs govern their attitudes and practices. Furthermore, the Consolidated Criteria for Reporting Qualitative Research checklist (COREQ) will be used to ensure rigorous development and reporting of qualitative protocols for this study (63).

## 3 Discussion

Gender plays a vital role in determining the health related behaviors in individuals and due to the gender based differences in socioeconomic and cultural aspects there is an inequitable exposure of modifiable risk factors leading to development of various chronic diseases. These differences are the reason why women are currently facing the biggest health threat from non-communicable diseases as evidenced by 65% deaths in the female population attributed to these diseases globally (50). The four highest contributors to 80% of the NCD related deaths are cancers, cardiovascular diseases, respiratory diseases and diabetes, and the probability of dying from any of these NCDs was higher in women than men in 164 countries (51). In Pakistan, females make up 53.61% of the total cancer cases, with most prevalent cancers being breast (38%), ovarian (6%), oral (4.9%), cervical (4%), and colorectal (3%) (52). A Pakistani study reported the prevalence of CVDs in women to be 18.3% as compared to 16.6% in men (53). In Pakistan, the prevalence for COPD as reported by a Karachi based study was found to be 13% in females as opposed to 5% in males. Females were also more likely to report COPD and respiratory distress symptoms than their male counterparts (54). According to the Non-Communicable Disease and Mental Health National Action Framework 2021–30, the burden of disease for diabetes in Pakistani women is 9.19% with Sindh province reporting the highest prevalence of diabetes in females (11.7%) (55).

Nutrition and physical activity interventions are now considered integral components of cancer management and reduce breast cancer incidence rates in both pre and post-menopausal women. A balanced diet and regular exercise help reduce the severity of cancer treatment side effects and decrease cancer related mortality in women (56). Additionally, obesity has been associated with a 35 to 40% higher risk of breast cancer recurrence and mortality. Weight gain both, before and after, breast cancer diagnosis results in poor prognosis and survival outcomes. However, a balanced diet, physical activity, and normal body weight positively impact survival rates and life quality in breast cancer patients (57). Lifestyle interventions including both nutrition and physical activity components can produce modest health impacts in as little as 6 weeks (18).

The Global Strategy on Diet, Physical Activity and Health, adopted by the World Health Assembly called for the immediate development and implementation of school based policies to promote good nutrition and physical activity (20). This highlights the need of lifestyle interventions involving teachers to improve the health status of both the educators and their students. Additionally, studies have found teachers to have a higher prevalence of unhealthy lifestyles and overall a poorer health status. This may be due to the excessive burn out and commitment that comes with the responsibility of educating a heterogeneous group of students. All these factors increase their susceptibility to develop accumulate lifestyle risk factors and later on chronic diseases (58). Furthermore, poor health in teachers then leads to increased absenteeism and even early retirement as the quality of life further deteriorates. Both physical health and work duties are impacted, leading to increased work dissatisfaction and overall decline in quality of life (59).

However, lifestyle interventions are influenced by various barriers and facilitators that influence their effectiveness. At the individual level, body image, self-regulatory skills, and a perceived sense of health and wellbeing, all contribute to adopting healthy diet and exercise. Whereas, time constraints and prioritizing other life aspects such as work and family obligations are the most reported hindrances. At the environmental level, social support and resources, act as barriers and facilitators depending on their absence and presence. Thirdly, the type of intervention and its mode of administration also impact the level of adherence, as participants are more likely to not follow dietary and exercise advice in the absence of regular interaction with experts and lack of support for behavior change (60). Another study implemented lifestyle interventions at the workplace and then interviewed participants about their experience. An important theme that emerged from lifestyle interventions at the workplace was ‘health as a barrier’ which participants described that their health problems such as NCDs were a barrier to their efficiency in work performance. However, they reported a positive impact of the lifestyle intervention and it led to a significant decrease in their health related stress, thereby also improving their work performance (61). Furthermore, reinforcement in the form of feedback and motivation to continue with the behavior changes despite not seeing immediate results, also increases adherence to behavior change strategies. Participants reported long-term impacts and social support in the form of group interventions to have a more positive impact in following a lifestyle intervention (62).

The proposed study has several limitations that need to be acknowledged. Firstly, purposive sampling of urban school may introduce sampling bias, thereby limiting generalizability of results beyond urban, middle-income employed females. Secondly, while baseline data will be collected for both control and intervention groups to establish baseline equivalence, the lack of multiple data collection points may weaken the parallel trends assumption. While multiple regression modeling and where needed, propensity score weighting will be employed to adjust for noticeable differences, the potential for residual confounding still exists. Thirdly, while anthropometric indicators will provide significant insights into participant NCD risk profile, lack of biochemical indicators may limit the study’s ability to assess subclinical physiological changes post-intervention. Fourth, the reliance on self-reported KAP measures may include social desirability bias, particularly in the intervention group after exposure to educational material. Lastly, steps will be taken to standardize intervention delivery and fidelity monitoring, variation in participant engagement may affect consistency of intervention outcomes.

## 4 Ethics and dissemination

Formal ethical approval will be obtained from the university’s review board and from the Federal Education Directorate (FDE) to carry out the study. Informed consent of the teachers prior to the study will be taken, after they have been properly briefed about the purpose, benefits and risks of the study. They will be informed of their choice to withdraw or refuse the study at any stage. If any participant refuses to take part in the study or drops out at any stage, she will bear no consequences and no pressure to continue the study. Anonymity of each participant will be ensured. Prior to conducting anthropometric assessment, each procedure will be explained to the participants to reduce any physical or psychological discomfort. Verbal consent from each participant will be taken before any anthropometric measurements. Same gender researcher will carry out all physical measurements. The anthropometric measurements will be take in a private and secluded room, where no one other than the participant and researcher will be present at the time of the measurement. The anthropometric readings will not be communicated to anyone other than the participant. The participants’ comfort, dignity, and religious and cultural sensitivity will be prioritized. No questionnaire form will contain the participant’s name or any other identification information such as home address or identity card numbers. Privacy and confidentiality of data shall be maintained. No one other than the researchers and analyst will have access to the study data. The key findings of the study will be communicated with relevant stakeholders via various social media platforms. Furthermore, the results will be published in peer-reviewed journals and at relevant national and international conferences.

## 5 Conclusion

This study protocol highlights the need for lifestyle changes in female teachers; a population sub-group that is often overlooked in health promotion and disease prevention efforts. The findings from our study are expected to inform public health and education policy makers regarding critical areas of intervention to reduce NCD risk among working females. This study aims to ultimately incorporate national health priorities within school-based wellness initiatives and bring forward female teachers as agents for change within their communities. The study findings will be helpful in designing future health policies and targeted lifestyle interventions that will have the potential to be scaled up at the institutional level. The study aims to support a coordinated and sustainable approach to female health promotion within the workplace.

## References

[1] Biswas T Townsend N Huda MM Maravilla J Begum T Pervin S . Prevalence of multiple non-communicable diseases risk factors among adolescents in 140 countries: a population-based study. EClinicalMedicine. (2022) 52:101591. doi: 10.1016/J.ECLINM.2022.101591, PMID: 36016694 PMC9396043

[2] Ong KL Stafford LK McLaughlin SA Boyko EJ Vollset SE Smith AE . Global, regional, and national burden of diabetes from 1990 to 2021, with projections of prevalence to 2050: a systematic analysis for the global burden of disease study 2021. Lancet. (2023) 402:203–34. doi: 10.1016/S0140-6736(23)01301-6, PMID: 37356446 PMC10364581

[3] Zhou B Carrillo-Larco R Danaei G Lancet LR-T, (2021) Worldwide trends in hypertension prevalence and progress in treatment and control from 1990 to 2019: a pooled analysis of 1201 population-representative 957–980. Available online at: https://www.thelancet.com/journals/lancet/article/PIIS0140-6736(2101330-1/fulltext [Accessed November 27, 2023] 10.1016/S0140-6736(21)01330-1 PMC8446938 34450083

[4] Sun J Qiao Y Zhao M Magnussen CG Xi B. Global, regional, and national burden of cardiovascular diseases in youths and young adults aged 15–39 years in 204 countries/territories, 1990–2019: a systematic analysis of global burden of disease study 2019. BMC Med. (2023) 21:222. doi: 10.1186/S12916-023-02925-4, PMID: 37365627 PMC10294522

[5] Menon GR Yadav J John D. Burden of non-communicable diseases and its associated economic costs in India. Soc Sci Humanit Open. (2022) 5:100256. doi: 10.1016/J.SSAHO.2022.100256

[6] Kazmi T Nagi MLF Razzaq S Hussnain S Shahid N Athar U. Burden of noncommunicable diseases in Pakistan. East Mediterr Health J. (2022) 28:798–804. doi: 10.26719/EMHJ.22.083, PMID: 36515443

[7] Gbadamosi MA Tlou B. Modifiable risk factors associated with non-communicable diseases among adult outpatients in Manzini, Swaziland: a cross-sectional study. BMC Public Health. (2020) 20:665. doi: 10.1186/S12889-020-08816-0, PMID: 32398061 PMC7216325

[8] Chowdhury SR Islam MN Sheekha TA Kader SB Hossain A. Prevalence and determinants of noncommunicable diseases risk factors among reproductive-aged women: findings from a nationwide survey in Bangladesh. PLoS One. (2023) 18:e0273128. doi: 10.1371/JOURNAL.PONE.0273128, PMID: 37294806 PMC10256164

[9] Bista B Dhungana RR Chalise B Pandey AR. Prevalence and determinants of noncommunicable diseases risk factors among reproductive aged women of Nepal: results from Nepal demographic health survey 2016. PLoS One. (2020) 15. doi: 10.1371/JOURNAL.PONE.0218840, PMID: 32176883 PMC7075700

[10] Lomia N Berdzuli N Pestvenidze E Sturua L Kereselidze NSM Topuridze M . Socio-demographic determinants of mortality from chronic noncommunicable diseases in women of reproductive age in the republic of Georgia: evidence from the national reproductive age mortality study (2014). Int J Women's Health. (2020) 12:89–105. doi: 10.2147/IJWH.S235755, PMID: 32161506 PMC7051896

[11] Khowaja PA Naz F Khowaja N Hussain SM Samad A Hayee A . The burden of non-communicable diseases in middle age population of Karachi, Pakistan. Pak J Med Health Sci. (2022) 16:97–7. doi: 10.53350/PJMHS2216397

[12] Idris IB Azit NA Abdul Ghani SR Syed Nor SF Mohammed Nawi A. A systematic review on noncommunicable diseases among working women. Ind Health. (2021) 59:146–60. doi: 10.2486/INDHEALTH.2020-0204, PMID: 33551443 PMC8365870

[13] Singh SK Chauhan K Puri P. Chronic non-communicable disease burden among reproductive-age women in India: evidence from recent demographic and health survey. BMC Womens Health. (2023) 23:20. doi: 10.1186/S12905-023-02171-Z, PMID: 36650531 PMC9843940

[14] Fagherazzi G Gusto G Fatouhi D El Mancini FR Balkau B Boutron-Ruault MC ., Mentally tiring work and type 2 diabetes in women: a 22-year follow-up study Eur J Endocrinol (2019) 180 257–263 doi: 10.1530/EJE-18-0804 PMID: 30840582

[15] Al-Hamdan R Avery A Salter A Al-Disi D Al-Daghri NM McCullough F. Identification of education models to improve health outcomes in Arab women with pre-diabetes. Nutrients. (2019) 11. doi: 10.3390/NU11051113, PMID: 31109110 PMC6566809

[16] Lagerweij GR Brouwers L De Wit GA Moons KGM Benschop L Maas AHEM . Impact of preventive screening and lifestyle interventions in women with a history of preeclampsia: a micro-simulation study. *Eur*. J Prev Cardiol. (2020) 27:1389–99. doi: 10.1177/2047487319898021, PMID: 32054298

[17] Fazrina G Retno U Hastuti B Adriani RB. Meta-analysis: the effect of lifestyle interventions on decreased postpartum weight retention. J Matern Child Health. (2023) 8:264–77. doi: 10.26911/THEJMCH.2023.08.03.02

[18] Kavanagh R Cooper R Kavanagh J Bolton L Keaver D. A pilot 6-week lifestyle intervention in women aged 50+ in Ireland. Phys Act Heal. (2022) 6:180–8. doi: 10.5334/paah.195

[19] Örs M. Healthy lifestyle behaviors among teachers working in public primary schools and affecting factors. Front Public Health. (2024) 12. doi: 10.3389/FPUBH.2024.1382385, PMID: 38645443 PMC11026594

[20] Hill J Draper CE de Villiers A Fourie JM Mohamed S Parker WA . Promoting healthy lifestyle behaviour through the life-orientation curriculum: teachers’ perceptions of the HealthKick intervention. S Afr J Educ. (2015) 35:1–9. doi: 10.15700/201503070003

[21] Ervasti J Kivimäki M Pentti J Salmi V Suominen S Vahtera J . Work-related violence, lifestyle, and health among special education teachers working in Finnish basic education. J Sch Health. (2012) 82:336–43. doi: 10.1111/J.1746-1561.2012.00707.X, PMID: 22671950

[22] Bogaert I De Martelaer K Deforcheb B Clarysb P Zinzen E. The physically active lifestyle of Flemish secondary school teachers: a mixed-methods approach towards developing a physical activity intervention. Health Educ J. (2015) 74:326–39. doi: 10.1177/0017896914536376

[23] Ak J O Im T Mm A. Impact of intervention on knowledge and risk factors of coronary heart disease among teachers in Sokoto, Nigeria. Int J Med Med Sci Full Length Res Pap. (2013) 5:476–88. doi: 10.5897/IJMMS2013.0983

[24] Karmakar A Bhattacharyya A Biswas B Dasgupta A Bandyopadhyay L Paul B. Effect of educational intervention on risk factors of cardiovascular diseases among school teachers: a quasi-experimental study in a suburb of Kolkata, West Bengal, India. BMC Public Health. (2023) 23:2304. doi: 10.1186/S12889-023-17227-W, PMID: 37990176 PMC10664257

[25] Parker EA Feinberg TM Lane HG Deitch R Zemanick A Saksvig BI . Diet quality of elementary and middle school teachers is associated with healthier nutrition-related classroom practices. Prev Med Rep. (2020) 18. doi: 10.1016/J.PMEDR.2020.101087, PMID: 32309116 PMC7155219

[26] Deslippe AL Soanes A Bouchaud CC Beckenstein H Slim M Plourde H . Barriers and facilitators to diet, physical activity and lifestyle behavior intervention adherence: a qualitative systematic review of the literature. Int J Behav Nutr Phys Act. (2023) 20:14. doi: 10.1186/S12966-023-01424-2, PMID: 36782207 PMC9925368

[27] Skoglund G Nilsson BB Olsen CF Bergland A Hilde G. Facilitators and barriers for lifestyle change in people with prediabetes: a meta-synthesis of qualitative studies. BMC Public Health. (2022) 22:553. doi: 10.1186/S12889-022-12885-8, PMID: 35313859 PMC8935766

[28] Andersen W Linge AD Jensen C. What works? A qualitative study of participants experiences of a traditional lifestyle intervention with a work focus. Int J Qual Stud Health Well-being. (2022) 17. doi: 10.1080/17482631.2022.2116988, PMID: 36053211 PMC9448365

[29] Ku CW Leow SH Ong LS Erwin C Ong I Ng XW . Developing a lifestyle intervention program for overweight or obese preconception, pregnant and postpartum women using qualitative methods. Sci Reports. (2022) 12:2511. doi: 10.1038/s41598-022-06564-2, PMID: 35169236 PMC8847557

[30] Schmidt SK Hemmestad L Macdonald CS Langberg H Valentiner LS. Motivation and barriers to maintaining lifestyle changes in patients with type 2 diabetes after an intensive lifestyle intervention (the U-TURN trial): a longitudinal qualitative study. Int J Environ Res Public Health. (2020) 17:1–16. doi: 10.3390/IJERPH17207454 PMC7602059 33066239

[31] Wasti SP Simkhada P Teijlingen ER van Sathian B Banerjee I The growing importance of mixed-methods research in health Nepal J Epidemiol (2022) 12:1175 doi: 10.3126/NJE.V12I1.43633 PMID: 35528457 PMC9057171

[32] Huang HC Szwerinski NK Nasrallah C Huang Q Chopra V Venditti EM . Lifestyle change program engagement in real-world clinical practice: a mixed-methods analysis. Transl Behav Med. (2023) 13:168–82. doi: 10.1093/TBM/IBAC098, PMID: 36694916 PMC10068905

[33] Komasawa M Yuasa M Shirayama Y Sato M Komasawa Y Alouri M. Impact of the village health center project on contraceptive behaviors in rural Jordan: a quasi-experimental difference-in-differences analysis. BMC Public Health. (2019) 19. doi: 10.1186/S12889-019-7637-9, PMID: 31664981 PMC6820982

[34] Wing C Simon K Bello-Gomez RA. Designing difference in difference studies: best practices for public health policy research. Annu Rev Public Health. (2018) 39:453–69. doi: 10.1146/ANNUREV-PUBLHEALTH-040617-013507/CITE/REFWORKS 29328877

[35] Thuy Duyen N Van Minh H Van Huy N Bao Giang K Thu Ngan T Xuan Long N ., Patterns of behavioral risk factors for non-communicable diseases in Vietnam: a narrative scoping review Health Psychol Open (2020) 7 doi: 10.1177/2055102920967248 PMID: 33173590 PMC7588771

[36] Budreviciute A Damiati S Sabir DK Onder K Schuller-Goetzburg P Plakys G . Management and prevention strategies for non-communicable diseases (NCDs) and their risk factors. Front Public Health. (2020) 8. doi: 10.3389/FPUBH.2020.574111, PMID: 33324597 PMC7726193

[37] Hewage N Wijesekara U Perera R. Determining the best method for evaluating obesity and the risk for non-communicable diseases in women of childbearing age by measuring the body mass index, waist circumference, waist-to-hip ratio, waist-to-height ratio, a body shape index, and hip index. Nutrition. (2023) 114:112135. doi: 10.1016/J.NUT.2023.112135, PMID: 37453224

[38] Bramhankar M Pandey M Rana GS Rai B Mishra NL Shukla A. An assessment of anthropometric indices and its association with NCDs among the older adults of India: evidence from LASI Wave-1. BMC Public Health. (2021) 21:1357. doi: 10.1186/S12889-021-11421-4, PMID: 34238276 PMC8268209

[39] Palinkas LA Horwitz SM Green CA Wisdom JP Duan N Hoagwood K. Purposeful sampling for qualitative data collection and analysis in mixed method implementation research. Admin Pol Ment Health. (2015) 42:533–44. doi: 10.1007/S10488-013-0528-Y, PMID: 24193818 PMC4012002

[40] Ames H Glenton C Lewin S. Purposive sampling in a qualitative evidence synthesis: a worked example from a synthesis on parental perceptions of vaccination communication. BMC Med Res Methodol. (2019) 19:26. doi: 10.1186/S12874-019-0665-4, PMID: 30704402 PMC6357413

[41] Shrivastava U Fatma M Mohan S Singh P Misra A. Randomized control trial for reduction of body weight, body fat patterning, and cardiometabolic risk factors in overweight worksite employees in Delhi, India. J Diabetes Res. (2017) 2017:1–12. doi: 10.1155/2017/7254174, PMID: 29318159 PMC5727835

[42] Yenit MK Kolbe-Alexander TL Gelaye KA Gezie LD Tesema GA Abebe SM . An evaluation of community health workers’ knowledge, attitude and personal lifestyle behaviour in non-communicable disease health promotion and their association with self-efficacy and NCD-risk perception. Int J Environ Res Public Health. (2023) 20:5642. doi: 10.3390/IJERPH20095642/S1 37174162 PMC10178727

[43] Ithnin M Mohamad nor N‘AU Juliana N Mohd Effendy N Sahar MA Abang Abdullah KH . Knowledge, attitudes and practices on risk factors of non-communicable diseases (NCDs): a cross-sectional survey among urban and rural adults in Negeri Sembilan, Malaysia. Int J Health Promot Educ. (2021) 59:236–46. doi: 10.1080/14635240.2020.1749526

[44] Cheza A Tlou B. Knowledge and perceptions about non-communicable diseases by people living with HIV: a descriptive cross-sectional study from Chitungwiza central hospital Zimbabwe. Afr Health Sci. (2022) 22:443. doi: 10.4314/AHS.V22I4.50 PMC10117497 37092054

[45] Demaio AR Dugee O Amgalan G Maximenco E Munkhtaivan A Graeser S . Protocol for a national, mixed-methods knowledge, attitudes and practices survey on non-communicable diseases. BMC Public Health. (2011) 11:1. doi: 10.1186/1471-2458-11-961 22208645 PMC3280340

[46] Subramaniam M Devi F AshaRani PV Zhang Y Wang P Jeyagurunathan A . Barriers and facilitators for adopting a healthy lifestyle in a multi-ethnic population: a qualitative study. PLoS One. (2022) 17:e0277106. doi: 10.1371/JOURNAL.PONE.0277106, PMID: 36322596 PMC9629631

[47] Ellison C Struckmeyer L Kazem-Zadeh M Campbell N Ahrentzen S Classen S. A social-ecological approach to identify facilitators and barriers of home modifications. Int J Environ Res Public Health. (2021) 18:8720. doi: 10.3390/IJERPH18168720, PMID: 34444467 PMC8391256

[48] Xie C Zhang Z Zhang X Li Y Shi P Wang S. Effects of interventions on physical activity behavior change in children and adolescents based on a trans-theoretical model: a systematic review. BMC Public Health. (2025) 25:1–16. doi: 10.1186/s12889-025-21336-z 39966763 PMC11834675

[49] Borhani M Hosseini ZS Shahabodin N Mehri A Kiani M Abedi M. Empowering rural housewives in Iran: utilizing the transtheoretical model to increase physical activity. J Prev Med Public Health. (2024) 57:167. doi: 10.3961/JPMPH.23.457, PMID: 38374712 PMC10999308

[50] Varì R Scazzocchio B D’amore A Giovannini C Gessani S Masella R. Gender-related differences in lifestyle may affect health status. Ann Ist Super Sanita. (2016) 52:158–66. doi: 10.4415/ANN_16_02_06 27364389

[51] Bennett JE Stevens GA Mathers CD Bonita R Rehm J Kruk ME . NCD countdown 2030: worldwide trends in non-communicable disease mortality and progress towards sustainable development goal target 3.4. Lancet. (2018) 392:1072–88. doi: 10.1016/S0140-6736(18)31992-5, PMID: 30264707

[52] Ikram A Pervez S Khadim MT Sohaib M Uddin H Badar F . National Cancer Registry of Pakistan: first comprehensive report of Cancer statistics 2015-2019. J Coll Physicians Surg Pak. (2023) 33:625–32. doi: 10.29271/JCPSP.2023.06.625, PMID: 37300256

[53] Zubair F Nawaz SK Nawaz A Nangyal H Amjad N Khan MS. Prevalence of cardiovascular diseases in Punjab, Pakistan: a cross-sectional study. J Public Heal. (2018) 26:523–9. doi: 10.1007/S10389-018-0898-4/TABLES/5

[54] Ghani S Thaver I Mehboob M Rafique K Ashraf M. Prevalence and predictors of respiratory symptoms in Karachi, Pakistan. Rawal Med J. (2022) 47:868–71.

[55] Non-Communicable Diseases & Mental Health National Action Framework 2021–2030 | ICCP portal. Available online at: https://www.iccp-portal.org/non-communicable-diseases-mental-health-national-action-framework-2021-2030 [Accessed June 29, 2024]

[56] Jia T Liu Y Fan Y Wang L Jiang E. Association of healthy diet and physical activity with breast cancer: lifestyle interventions and oncology education. Front Public Health. (2022) 10. doi: 10.3389/FPUBH.2022.797794, PMID: 35400043 PMC8984028

[57] Cortesi L Sebastiani F Iannone A Marcheselli L Venturelli M Piombino C . Lifestyle intervention on body weight and physical activity in patients with breast cancer can reduce the risk of death in obese women: the EMILI study. Cancers (Basel). (2020) 12. doi: 10.3390/CANCERS12071709, PMID: 32605075 PMC7407899

[58] Wilf-Miron R Kittany R Saban M Kagan I Saban M. Teachers’ characteristics predict students’ guidance for healthy lifestyle: a cross-sectional study in Arab-speaking schools. BMC Public Health. (2022) 22:1. doi: 10.1186/s12889-022-13795-5 35883162 PMC9321300

[59] Coledam DHC De Arruda GA Ribeiro EAG Cantieri FP. Self-rated health among teachers: prevalence, predictors, and impact on absenteeism, presenteeism, and sick leave. Rev Bras Med Trab. (2021) 19:426. doi: 10.47626/1679-4435-2021-619, PMID: 35733536 PMC9162284

[60] de Jong M Jansen N van Middelkoop M. A systematic review of patient barriers and facilitators for implementing lifestyle interventions targeting weight loss in primary care. Obes Rev. (2023) 24:e13571. doi: 10.1111/OBR.13571, WGROUP:STRING:PUBLICATION 37226636

[61] Jones S Brown TJ Watson P Homer C Freeman C Bakhai C . Commercial provider staff experiences of the NHS low calorie diet programme pilot: a qualitative exploration of key barriers and facilitators. BMC Health Serv Res. (2024) 24:1–13. doi: 10.1186/s12913-023-10501-y 38200539 PMC10782528

[62] Kuriakose L Kuczynska P Timpel P Yakub F Bayley A Papachristou Nadal I. Effectiveness of behaviour change techniques on lifestyle interventions of patients with a high risk of developing cardiovascular disease. Using a qualitative approach. Health Soc Care Community. (2020) 28:998–1009. doi: 10.1111/HSC.12933, PMID: 31965675

[63] Jowsey T Deng C Weller J. General-purpose thematic analysis: a useful qualitative method for anaesthesia research. BJA Educ. (2021) 21:472. 34840819 10.1016/j.bjae.2021.07.006 PMC8606608

